# Application of a Dual Internally Quenched Fluorogenic Substrate in Screening for D-Arginine Specific Proteases

**DOI:** 10.3389/fmicb.2019.00711

**Published:** 2019-04-03

**Authors:** Andreas H. Simon, Sandra Liebscher, Tobias H. Aumüller, Dennis Treblow, Frank Bordusa

**Affiliations:** ^1^Institute of Biochemistry/Biotechnology, Charles Tanford Protein Centre, Martin-Luther-University Halle-Wittenberg, Halle, Germany; ^2^Max Planck Research Unit for Enzymology of Protein Folding, Halle, Germany

**Keywords:** D-amino acids, screening, proteases, internally quenched fluorogenic substrates, D-arginine, penicillin binding proteins, DD-peptidases

## Abstract

The application of D-stereospecific proteases (DSPs) in resolution of racemic amino acids and in the semisynthesis of proteins has been a successful strategy. The main limitation for a broader application is, however, the accessibility of suitable DSPs covering multiple substrate specificities. To identify DSPs with novel primary substrate preferences, a fast specificity screening method using the easily accessible internally quenched fluorogenic substrate aminobenzoyl-D-arginyl-D-alanyl-*p*-nitroanilide was developed. By monitoring both UV/*vis*-absorbance and fluorescence signals at the same time it allows to detect two distinct D-amino acid substrate specificities simultaneously and separately with respect to the individual specificities. In order to identify novel DSP specificities for synthesis applications, DSPs specific for D-arginine were of special interest due to their potential ability as catalysts for substrate mimetics-mediated peptide and protein ligations. D-alanine in the substrate served as positive control and reference based on its known acceptance by numerous DSPs. *In silico* analysis suggested that DSPs are predominantly present in gram-positive microorganisms, therefore this study focused on the bacilli strains *Bacillus thuringiensis* and *Bacillus subtilis* as potential hosts of D-Arg-specific DSPs. While protease activities toward D-alanine were found in both organisms, a novel and so far unknown D-arginine specific DSP was detected within the culture supernatant of *B. thuringiensis*. Enrichment of this activity via cation exchange and size exclusion chromatography allowed isolation and further characterization of this novel enzyme consisting of a molecular mass of 37.7 kDa and an enzymatic activity of 8.3 U mg^-1^ for cleaving the D-Arg|D-Ala bond in the detecting substrate. Independent experiments also showed that the identified enzyme shows similarities to the class of penicillin binding proteins. In future applications this enzyme will be a promising starting point for the development of novel strategies for the semisynthesis of *all*-L-proteins.

## Introduction

Proteases are ubiquitously distributed in all three domains of life and fulfill functions in various cellular and biological processes, nutrient acquisition and microbial defense processes (Kageyama, 2002; Lee et al., 2008; Zapun et al., 2008). In addition, proteolytic enzymes are widely used in industrial and research applications (Rai and Mukherjee, 2010; Fickers et al., 2011; Vandermarliere et al., 2013). Beside applications using their native hydrolysis activity, proteases are meanwhile also well established for synthesis processes that are based on their ability to catalyze the reverse of hydrolysis. Mild reaction conditions and high substrate stereospecifity as well as distinct selectivity compared to chemical processes make proteases also unique as ligation reagents for modification and semisynthesis of peptides and proteins (Bordusa, 2002a). Various strategies to broaden their application based on substrate mimetics, rational enzyme design as well as directed evolution toward alternative substrate-recognition or the discovery of novel enzymes with unique specificities were developed (Bordusa, 2002b; Clancy et al., 2010; Wang et al., 2012; Liebscher et al., 2014). Semisynthesis of native or selectively modified *all*-L-proteins catalyzed by alkaline D-peptidase, which is, e.g., highly specific toward D-Phe, gave rise to a completely new synthesis concept for proteins via enzymatic fragment condensation catalyzed by DSPs (Wehofsky et al., 2008). By this concept, the native *all*-L-protein Parvulin 10 was ligated with an overall yield of 61%, under the usage of a *4*-guanidino-phenylester (OGp) substrate mimetic, bearing in addition for the phenylalanin-imitating phenyl ring a specifity mediating guanidino group which is similar to the side chain of arginine. This similarity makes OGp esters perfect substrates for arginine-specific proteases. Furthermore, the direct use of D-amino acids within the sequence of antimicrobial agents or for pharmaceutical applications is also widely considered (Gaspar et al., 2013). However, purely chemical synthesis is expensive and often results in rather low overall yields of those multistep reactions. DD-peptidases (DSPs) (E.C. 3.4.16.4), originally catalyzing the hydrolysis of a peptide bond between two D-amino acid moieties, would have a significant potential as ligation catalyst in the semisynthesis of those biopolymers.

The main limitation for a broader application of DSPs relates to the limited range of distinct substrate specificities of these enzymes. Previously, D-amino acid accepting enzymes like D-aminoacylase, D-aminopeptidase or the ADP were described (Sugie and Suzuki, 1980; Asano et al., 1996; Matsushita-Morita et al., 2013). However, most of these enzymes are characterized only with a very limited number of suitable peptide substrates (Asano and Lubbehusen, 2000). Until now, only enzymes consisting of the group of PrP and their subgroub of DD-peptidases like the ADP were certainly shown to exhibit real peptidase activity including the cleavage of D-amino acid derived peptide bonds in respective substrates (Asano et al., 1996; Pratt, 2008). Remarkably, even within these enzyme groups the ADP from *Bacillus cereus* DF4-B was the only DD-peptidase showing endopeptidase activity toward D-phenylalanine-containing peptide substrates (Asano et al., 1996). This activity, in combination with its L-amino acid specific S’-subsites, make ADP unique for the semisynthesis of *all*-L-peptides/proteins via substrate mimetic assisted synthesis yielding product concentrations up to more than 90% for peptide and more than 60% for protein semisynthesis without the risk of any undesired product hydrolyses which are characteristic when classical L-amino acid specific proteases are used as ligation catalysts (Wehofsky et al., 2008; Nakano et al., 2015).

**FIGURE 1:**
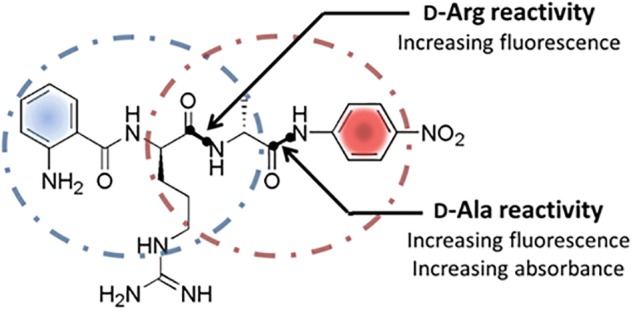
Structure and cleavage sites of the dual screening substrate Abz-D-Arg-D-Ala-*p*NA. Multiple proteolytic specificities toward D-Arg and/or D-Ala can be detected via fluorescence measurements at λ = 320 nm/λ = 420 nm and simultaneous UV/*vis*-spectroscopy at 405 nm. Positive fluorescence signals indicate a general cleavage activity toward D-Arg and/or D-Ala while positive UV/*vis*-absorbance signals display a proteolytic specificity for D-Ala exclusively.

Keeping the already proven enzymatic activities for the cleavage of D-amino acid derived substrates in mind, PrPs including DD-peptidases appear to be interesting natural sources of unknown DSPs in general and enzymes with novel substrate specifities in particular. PrPs are generally involved in bacterial cell wall biosynthesis as murein transpeptidases that catalyzes the formation of the peptide mesh-like network mediating the cell stability (Sauvage et al., 2008). Due to the known variances within the murein peptide sequences, a natural set of corresponding PrPs differing in their primary substrate specificities can be expected. Presently used screening systems in this respect focus mainly on the substitution of D-amino acids in media, the use of classical single amino acid-*p*NA-derivatives, D-aa containing short peptides like (D-Phe)_4_ and classical FRET (Förster Resonance Energy Transfer) substrates (Asano and Lubbehusen, 2000; Kaman et al., 2011, 2013). General drawbacks are long incubation times ranging from hours to days, limited substrate solubility, time consuming quantification of the reactions and at least in some cases, complex reaction analysis. Making the identification of DSPs with novel D-amino acid specificities more efficient, the function of a internally quenched fluorogenic substrate (IQFS) with the dual use as chromogenic substrate was evaluated. In addition the intended substrate allows a site-specific determination of hydrolytic events toward a distinct amino acid without the need of an ongoing MS analysis step of the peptides substrate. Classical IQFSs normally consist of a fluorescence donor group at one terminal end of the substrate and a quenching acceptor counterpart at the other (Hirata et al., 1995). The general function of the conventional IQFSs for determining the enzyme activity as well as individual substrate specificty was already succesfully demonstrated for numerous classical L-amino acid specific proteases (Chagas et al., 1995; Pimenta et al., 2001). In this contribution, the ability of the dual fluoro- and chromophoric IQFS Abz-D-Arg-D-Ala-*p*NA for the identification of a novel DSP with a so far unknown D-Arg specificity was investigated. The dual IQFS conceptionally bears an 2-Abz functionality at its *N*-terminus serving as the fluorescence donor group which is combined with the chromophoric *p*-nitroaniline (*p*NA) located at the *C*-terminal end of the substrate (Figure 1). In addition to its chromophoric properties, the *p*NA-moiety simultaneously acts as fluorescence acceptor group for the Abz-donor resulting in a quenched fluorescence signal of the intact substrate. Any proteolytic events toward D-Arg and/or D-Ala within the substrate go along with a respective fluorescence signal (λ = 320 nm, λ = 420 nm). At the same time, analysis of the liberated *p*NA group via simultaneous *vis*-spectroscopy (λ 405 nm) enables the detection of enzyme activities toward D-Ala exclusively. In this case study we used the advantages of robustness, sensitivity, and specificity of the approach to identify and isolate novel D-arginine specific hydrolases.

## Materials and Methods

### Synthesis of Abz-D-Arg-D-Ala-*p*NA

Amino acid derivatives, coupling reagents, media components, and chemical compounds were obtained from Bachem (Switzerland), Merck (Germany), Roth (Germany), and Fluka (Germany). All reagents were of the highest commercial purity. If necessary, solvents and reagents were purified and/or dried by standard procedures. The synthesis of the internally quenched fluorescence substrate was performed by standard coupling, purification and deprotection methods as depicted in Supplementary Figure S1 (details see supporting information). Preparative separations were performed by HPLC using a RP C18 column (VYDAC^^®^^ Protein & Peptide C18) via gradient elution with water/acetonitrile (v/v) ranging from 20 to 80% ACN containing 0.1% trifluoroacetic acid at room temperature and flow rates of 10 ml min^-1^. Product elution was followed at 220 nm. The overall product yield of the whole synthesis was 36%. The identity and purity of the product was determined by mass spectrometry [m/z_calc._ = 484; m/z_found_ = 485 (M+H^+^)] and analytical HPLC at 220 nm, respectively (see Supplementary Figure S2). The *enantiomeric excess (ee)* of the final peptide derivative was found to be higher 99% as proven by enzymatic resolution experiments using the L-Ala and L-Arg specific proteases elastase and trypsin, respectively. Resolution reactions were performed with 2 mM of the IQFS and 5 μM of the respective protease for 4 h. Within the detection limit of the fluorescence measurements, which determine concentrations ≥0.01 μM, no cleavage products could be detected indicating an *ee* higher than 99% for the IQFS synthesized. Analog reactions with the corresponding *all*-L-IQFS were performed as positive control resulting in a complete hydrolysis of the substrate.

### Bacillus Cell Fractionation

The gram-positive bacterial strains *Bacillus thuringiensis* Berliner 1915 and *Bacillus subtilis* subsp. *subtilis* were obtained from the German Collection of Microorganisms and Cell Cultures (DSMZ). *Bacillus* strains were grown in 50 ml NB-medium in 250 ml flasks under continuous shaking for 24 h at 30°C (LaPage et al., 1970). 100 ml of the particular *Bacillus* culture was taken and sonicated (5 min, amplitude 20%) for cell disruption and later analyzed as “culture fraction” reflecting the complete DSP activity originated by the cells. Remaining cells were sedimented at 4,000 ×*g* for 15 min, yielding 0.13 and 0.21 g/10 ml wet weight for *B. subtilis* and *B. thuringiensis*, respectively. The “culture supernatant fraction” was separated via decantation and the cells (‘whole cell fraction‘) were resuspended in ml resuspension buffer (10% w/v sucrose, 5 mM MgCl_2_, 1 mg ml^-1^ lysozyme, 50 mM Tris/HCl, pH 7.5). After incubating the suspension for 30 min at 37°C, the cells were spun down for 10 min at 10,000 ×*g* resulting in a two phase supernatant and a pellet. The upper phase of the supernatant was removed, representing the “cell wall fraction” and the remaining supernatant was discarded. The pelleted cells were resuspended in 1 ml of cold PBS and lysed by sonication (10 s pulses at 40% amplitude). After two further washing/centrifugation steps at 50,000 ×*g* for 30 min at 4°C and at 400,000 ×*g* for 45 min at 4°C the top 30% of the supernatant were kept as “cytoplasmic fraction.” The pellet was washed twice in PBS and centrifuged at 400,000 ×*g* for 45 min at 4°C. The suspended pellet in PBS was kept as “membrane fraction.” For a better comparability of the results, an amount corresponding to 1 mg wet weight of the original whole cell fraction paste was used within the initially activity measurements.

### Purification and Identification of D-Arg Specific DSP

*Bacillus thuringiensis* was grown in a total volume of 14 liters of NB-medium with continuous shaking at 30°C for 24 h. After separating the cells by centrifugation at 5,000 ×*g* for 30 min, the supernatant proteins were enriched using a two-step chromatographic strategy at 8°C using phosphate buffer (0.1 M potassium phosphate buffer, pH 8.0, and 0.1 M NaCl). For this purpose, the supernatant was loaded on a Toyopearl^^®^^ Supelco SP 650 M column and eluted with a single step gradient to 0.4 M NaCl. Fractions containing the desired enzymatic activity were identified using IQFS, pooled, concentrated, and dialyzed against the original phosphate buffer. The last step of purification represents a size exclusion chromatography (HiLoad 16/60 Superdex^TM^ 75 prep grade column; phosphate buffer 1 ml min^-1^). D-Arg DSP containing fractions were combined, concentrated, and analyzed via SDS-polyacrylamide gel electrophoresis and mass spectrometry.

### Analytical Methods

Distribution analysis of DSPs in putative host organisms was carried out using the database eggNOG3.0 (Jensen et al., 2008). The protein sequence of *B. cereus* Alkaline D-Peptidase was used as template (Asano et al., 1996).

Enzyme activities were determined by measuring the rate of hydrolysis using a NOVOstar microplate reader (BMG Laboratories, Germany) and clear-bottom 96-well plates (Corning, United States). For fluorescence analysis an excitation wavelength of 320 nm and an emission wavelength of 420 nm were used. Simultaneously, released *p*NA was detected at an absorption wavelength of 405 nm (see Supplementary Figure S5). All measurements were performed in phosphate buffered saline (PBS) or PBS-buffered fractions pH 7.4. The substrate concentrations varied between 100 to 400 μM. The enzymatic reactions were followed for time intervals between 2 and up to 24 h depending on the individual enzyme activity. Traces of spontaneous hydrolysis of the substrate were measured in parallel and used for calibration. One unit of enzymatic activity (1 U) is defined as the conversion of 1 μmol IQFS per min. After determining the enzyme activity via fluorescence and absorbance measurements, the samples were analyzed via analytical reversed-phase HPLC. For this purpose, the reactions were quenched with 50% (v/v) acetic acid and subsequently analyzed using a C18 column (ACQUITY UPLC^^®^^ BEH130). Elution was performed with an acetonitrile/water gradient from 0 to 40% (v/v) ACN containing 0.05% trifluoroacetic acid at a flow rate of 0.5 ml min^-1^. Multiple wavelengths for detection were used simultaneously (220, 254 and 320 nm). For LC-MS analysis a RP C18 column (X-Bridge^TM^ BEH300), gradient elution and a Waters micromass ZQ detector were used.

## Results

### Prediction of Putative DSP Host Organisms

**FIGURE 2:**
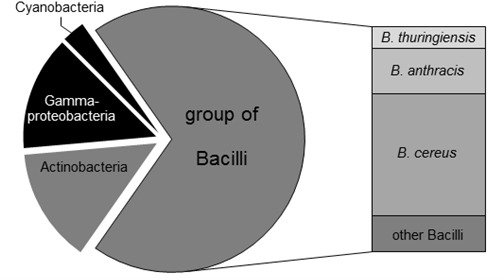
Distribution of ADP-like enzymes in bacteria. Using the eggNOG3.0 database, 72 putative host organisms for DSPs were identified. 12 out of 72 organisms are gram-negative (colored in black). The remaining 60 gram-positive species (shown in gray) mainly relates to *Bacilli* strains (50 proteases). High populations of putative DSPs were predicted for pathogenic microorganisms like *B. cereus* (27 DSPs) and *B. anthracis* (10 DSPs). The less pathogenic organism *B. thuringiensis* is supposed to serve as host for five DSPs.

In order to identify potential host organisms encoding putative ADP-like DSPs with alternative D-amino acid specificities, bioinformatic homology studies were initially performed (Figure 2). For this purpose, ADP of *B. cereus* DF4-B was used as reference protein sequence. Using the eggNOG3.0 database (Jensen et al., 2008), a total number of 72 proteins were identified within the bacterial domain showing an orthology to the primary structure compared to ADP. 12 out of these 72 identified candidate proteins were found to be distributed in gram-negative bacteria (Figure 2, colored in black). Within the group of gram-positive bacteria, 10 candidate enzymes are encoded in actinomycetes whereas the remaining putative DSPs were identified in bacilli genomes (Figure 2, colored in gray). Due to its limited toxicity and pathogenity the *bacillus* strain *B. thuringiensis* was chosen for further experimental estimation. As predicted by the bioinformatic study, the genome of *B. thuringiensis* encodes 5 ADP-like enzymes according to the confidence interval of sequence homology. As negative control, *B. subtilis* was additionally selected. The latter represents a well characterized organism that misses any ADP-like DSPs encoding PbP/PrP enzymes exclusively, which are supposed to have a strict D-Ala specificity (Popham et al., 1995; Sassine et al., 2017).

### Analysis of Predicted DSP Activities in *B. thuringiensis* and *B. subtilis*

Hydrolytic events toward the IQFS were monitored by real-time measuring of changes in fluorescence and absorbance signals, simultaneously. According to the dual nature of the substrate, positive fluorescence signals indicate any proteolytic substrate cleavages after both D-Ala and/or D-Arg in a general manner. Discrimination between both individual specificities can be made via simultaneous UV/*vis*-absorbance spectroscopy recording the liberation of the *p*NA-group at 405 nm indicating the specific cleavage of the D-Ala-*p*NA-bond exclusively.

**FIGURE 3:**
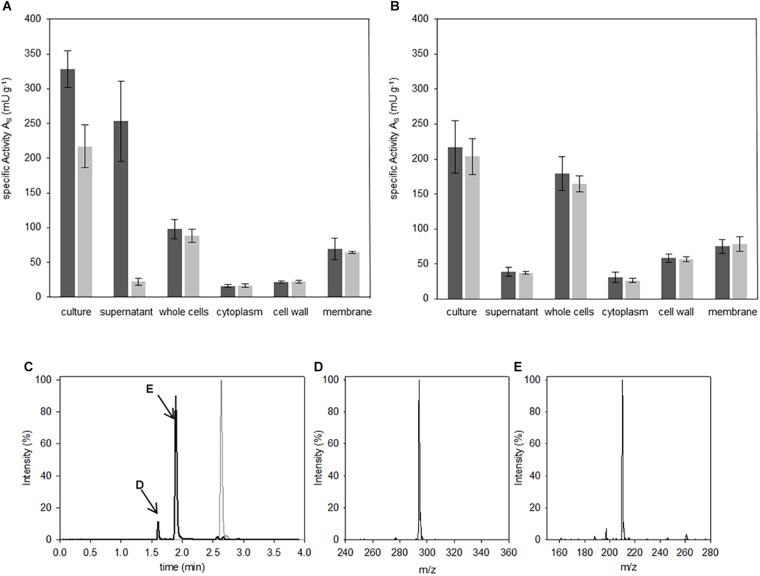
Localization of Abz-D-Arg-D-Ala-*p*NA activity. Hydrolysis studies were performed in fractions of *B. thuringiensis* **(A)** and *B. subtilis* **(B)**. 400 μM IQFS were incubated at 30°C and changes in fluorescence (dark gray) and UV/*vis*-absorption (light gray) were monitored for 8 h (wavelength fluorescence: λ = 320 nm/λ = 420 nm, wavelength UV/*vis*-spectroscopy: λ = 405 nm). Chromatographic reaction analysis of the supernatant fraction of *B. thuringiensis* analyzed via HPLC **(C)** (gradient elution: 0–40% ACN, λ = 320 nm). In comparison to the intact starting IQFS (gray), the formation of two product peaks were detected (black line, peaks are indictated with D and E respectivly). The corresponding products were identified via mass spectrometry as Abz-D-Arg-OH **(D)** [m/z_found_ = 294.3 (M+H^+^); m/z_calc._ = 293.3] and NH_2_-D-Ala-pNA **(E)** [m/z_found_ = 210.2 (M+H^+^); m/z_calc._ = 209.2].

Biological probes from the two *Bacilli* strains were prepared and fractionated as described in the Materials and Methods section. Accordingly, volumes of 100 μl of each fractionation of the *Bacilli* cultures were incubated with 400 μM of the IQFS Abz-D-Arg-D-Ala-*p*NA for 8 h. Analyses of the culture containing cells together with the corresponding growth medium showed DSP activities in both *Bacilli* strains and toward both cleavage sites (Figure 3A,B). A more detailed comparison shows, however, differences in the individual fluorescence- and absorbance-based activities between the two *Bacilli* strains. In fact, the turnover rate of the substrate in the case of *B. thuringiensis* culture fraction showed a higher fluorescence-related activity (327 mU g^-1^) compared to that corresponding to the absorption (217 mU g^-1^). From this disparity one can conclude a bias in the substrate specificity toward the cleavage after D-Arg over D-Ala. In contrast, no difference between both signals was found for *B. subtilis* (fluorescence-based activity 219 mU g^-1^ vs. absorption-based activity 203 mU g^-1^). Analysis of the whole cell fraction of *B. subtilis* (*cf.* Figure 3B) showed that the DSP-activity measured by both fluorescence and absorbance spectroscopy may distributed mainly between the fractions of the cell wall (58 mU g^-1^) and the membrane (74 mU g^-1^). Related to the latter, nearly the same DSP-activity was found within the membrane fraction of *B. thuringiensis* (69 mU g^-1^), while the cell wall fraction showed less DSP-activity compared to *B. subtilis* (22 mU g^-1^). Strikingly, the most significant differences between the fluorescence- and absorbance-based activities (253 mU g^-1^ vs. 24 mU g^-1^, two-tailed *P*-value <0.0001) arise for the supernatant fraction of *B. thuringiensis.* This strongly indicates a unique D-Arg specific DSP, which is obviously not present in *B. subtilis*, accounting for the bias of both signals in the *B. thuringiensis* strain. To confirm the enzymatic cleavage of the D-Arg-D-Ala-bond as indicated by the dominant fluorescence signal, additional HPLC-MS analyses of the reaction mixture were performed (Figure 3C). The results clearly verify the formation of two reaction products which could identified as Abz-D-Arg-OH and NH_2_-D-Ala-*p*NA finally proving the presence of a D-Arg specific DSP in *B. thuringiensis*.

### Isolation and Initial Characterization of D-Arg-Specific DSP of *B. thuringiensis*

**FIGURE 4:**
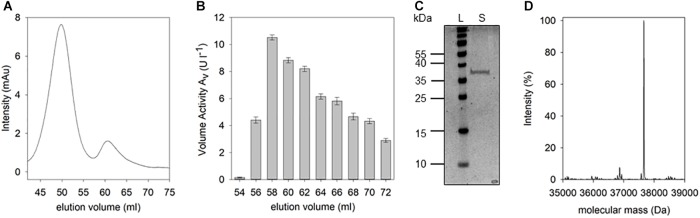
Isolation and purification of D-Arg specific DSP. The supernatant of *B. thuringiensis* was purified as described. After size exclusion chromatography **(A)** the volume activity within the fractions was determined via fluorescence measurements using IQFS **(B)** (200 μM IQFS, reaction time 10 min). After pooling and concentrating DSP-active fractions, the resulting sample was analyzed via SDS-PAGE **(C)** (12.5% SDS-PAGE, silver staining, PageRuler^TM^ Prestained Protein Ladder [L], concentrated fractions [S]). Mass spectrometic analysis of the purified protein reveals a molecular mass of the D-Arg specific DSP of 37,661 Da **(D)**.

Following the D-Arg DSP activity detected in the culture supernatant of *B. thuringiensis*, the responsible corresponding protein was purified. Pure protein could be obtained by initial cation exchange chromatography which was applied due to the assumption that the DSP of interest may belong to the class of DD-peptidases known for their basic isoelectric points. After optimization of elution conditions, fractions producing a fluorescence signal in presence of the IQFS were identified, pooled, dialyzed, and further purified by size exclusion chromatography (Figure 4A). At elution volumes of 54–72 ml the D-Arg specific enzyme could be detected with a maximum cleavage activity of 10.9 U l^-1^ at a retention volume of 58 ml (Figure 4B). After pooling and concentrating the corresponding fractions, the resulting protein sample was analyzed by SDS-PAGE showing a single protein band with a molecular mass of about 37 kDa (Figure 4C). The enzymatic activity of this final protein fraction was determined with 8.3 U mg^-1^ using the IQFS as substrate (see Supplementary Figure S3). MS-spectrometric analysis of the purified enzyme revealed a molecular mass of 37,661 Da (Figure 4D). Furthermore, the novel enzyme shows strong inhibition by PMSF (phenylmethane sulfonyl fluoride), which chemoselectively reacts with activated serine residues of the catalytic machinery of serine protease. In addition, the enzyme also behaves like a PrP showing the typical shift of its molecular mass upon addition of β-lactam antibiotics like ampicillin (see Supplementary Figure S4). Finally, *N*-terminal fragmentation analysis was performed (Mortz et al., 1996) revealing SSLQTSTQQSDR as the *N*-terminal amino acid sequence. Analytical BLAST of this primary sequence identified the enzyme as D-stereospecific hydrolase of *B. thuringiensis* (PMID: C3FM16) (Mount, 2007).

## Discussion

D-stereospecific Proteases still represent an underestimated class of proteases with only a very limited variety of distinct D-amino acid specificities. On the other hand, a significant quantity of applications in academic as well as applied research has been already developed which are, however, hampered by the only small number of suitable enzymes presently known. To broaden the scope of these interesting enzymes in this context, identification of novel DSPs with alternative D-amino acid specificities is an important approach. Regarding our own concerns, DSPs specific for D-Arg are of particular interest especially for their use as ligation catalysts in the synthesis and modification of native *all*-L-peptides and -proteins. DSPs with D-Arg specificity are, however, unkown so far. To avoid overlapping with a second, undesired substrate specificity, a novel dual IQFS substrate was developed enabling the analysis of two distinct amino acid specificities at the same time in a fast, robust and sensitive manner. Although classical IQFSs are already well established especially for the characterisation of L-stereospecific proteases (Oliveira et al., 2012), we could prove that the dual IQFS shows clear benefits over the classical analog which should not be restricted to the screening for novel DSP-activities in organisms. So far, DSPs were identified by either non-functionalized peptides like (D-Phe)_4_ or (D-Asp)_8_ or in combination with substrates bearing single chromogenic functionalities (Asano and Lubbehusen, 2000). Classical FRET substrates have been also applied in the context of DSPs mainly for profiling DSPs in order to classify organisms (Kaman et al., 2013). Besides this, Kaman showed that *Bacillus spp.* have the potential to cleave substrates consisting of two D-amino acids such as D-Lys-D-Lys, D-Leu-D-Leu and D-Arg-D-Arg (Kaman et al., 2013). Whilst being able to prove a similar activity in the supernatant of *B. thuringiensis* within hours, the used IQFS was also utilized to isolate and finally characterize novel DSP. Remarkably the last step can be performed within minutes and demonstrating the high potential of this substrate class for characterizing DSPs. In contrast, the dual IQFS used in our study has the advantage to screen for at least two distinct substrate specificities in only one single screening step simultaneously. Beside its sensitifity, flexibility and simplicity in application, the use of the substrate is rather pH independent enabling screenings at variuos pH-values ranging from pH 4 to 9 (Tanojo et al., 1997). Based on the broad range of pH, the approach is likewise suitable for screening microorganisms ranging from acidophiles to basidophiles. Such microorganisms represent, however, an interesting pool of putative DSPs due to occurrence of alternative metabolism pathways or D-amino acid containing peptides and proteins in the microbiome.

The detection of DSPs specific for D-Ala within the investigated organisms can be correlated to the occurrence of PrPs and PbPs within that group of *Bacilli*, which are already known to exhibit D-Ala protease activity (Blumberg and Strominger, 1974; Matsuhashi et al., 1979; Pratt and McLeish, 2010). It is further known that such activities correlate with the role of these enzymes in cell wall biosynthesis. Accordingly, the highest concentration of those enzymes can be found in the cell growth phases of the *Bacilli* (Popham and Young, 2003; Mattei et al., 2010). Keeping this in mind, the *Bacilli* strains used were fractionated in their late exponential growth phase going along with the finding that the highest D-Ala proteolytic activity was detected in the membrane and cell wall fractions. In contrast, the additional proteolytic activity toward D-Arg was found in the supernatant of the culture of *B. thuringiensis* indicating a biological function of the novel DSP distinct from classical PrPs and PbPs. Interestingly, D-aminopeptidase from *O. anthropoi* as well as ADP from *B. cereus*, two enzymes with lower similarity to PrPs and PbPs, were also isolated from the supernatant of the respective cell cultures (Asano et al., 1996; Fanuel et al., 1999). From a more practical point of view, the identified D-Arg specific DSP adds a novel D-amino acid specificity to the known repertoire of D-stereospecific proteases which might be suitable for application in protein semisynthesis or racemic resolution. Studies in this direction are presently underway.

## Author Contributions

TA, AS, and SL conceived of the present idea. TA and AS carried out the chemical synthesis. Bioinformatic studies and biological assays were carried out by AS. All authors discussed the results and contributed to the final manuscript. AS, TA, and SL wrote the manuscript with support from FB. FB supervised the project.

## Conflict of Interest Statement

The authors declare that the research was conducted in the absence of any commercial or financial relationships that could be construed as a potential conflict of interest.

## Abbreviations

Abz: aminobenzoic acid
ADP: Alkaline D-Peptidase
DSP: D-stereospecific Protease
IQFS: internally quenched fluorogenic substrate
PbP: Penicillin binding Protein
*p*NA: *para*-nitroanilide
PrP: Penicillin recognizing Protein
