# Supplementary material for: Application of a Dual Internally Quenched Fluorogenic Substrate in Screening for D-Arginine Specific Proteases
Source: Front Microbiol. 2019 Apr 3;10:711. doi: 10.3389/fmicb.2019.00711 (PMC6456654; doi:10.3389/fmicb.2019.00711)
Supplement: Supplementary file 1 [file Data_Sheet_1.PDF]

Supplementary Material

Application of a Dual Internally Quenched Fluorogenic Substrate in Screening for D-Arginine Specific Proteases

Andreas H. Simon, Sandra Liebscher, Tobias H. Aumüller, Dennis Treblow and Frank Bordusa*

***Correspondence:** frank.bordusa@biochemtech.uni-halle.de

1 Supplementary Methods

Synthesis of Abz-D-Arg-D-Ala-*p*NA

A general synthesis scheme of Abz-D-Arg-D-Ala-*p*NA is depicted in supplementary figure 1. In the first step of reaction the building block H-D-Ala-*p*NA was synthesized by coupling Boc-D-Ala-OH and 4-nitroaniline (*p*NA) via the method of mixed anhydrides using isobutyl chloroformate (IBCF) as coupling reagent (Figure S1, step A) (Anderson et al. 1967; König and Geiger, 1970). For this purpose, one equivalent (eq.) Boc-D-Ala-OH was activated by adding 1.1 eq. IBCF and 4 eq. of *N*-ethyl morpholine (NEM) at -20°C. After 15 min preincubation the coupling reaction was started by adding 1.2 eq. of recrystallized *p*NA (Baumann, 1979). The reaction mixture was incubated for 2 hours at -15°C and subsequently for another 12 hours at room temperature. The reaction progress was followed by thin layer chromatography (TLC). After complete conversion the reaction was stopped by addition of water. The organic phase was extracted with 5% (w/v) $KHSO_4$ solution, followed by saturated $NaHCO_3$ and saturated NaCl solution. Afterwards the organic phase was dried over Na_2SO_4. Finally, the solvent was removed by vaporization. Removal of the Boc-protecting group occurred by adding of 95% (w/v) trifluoracetic acid (TFA) (Figure S1, step B) (Lundt et al., 1978). After vaporizing TFA, the remaining destillate was dissolved in acetonitrile (ACN)/water and purified via preparative HPLC. Product containing fractions were pooled and freeze dried. The final H-D-Ala-*p*NA was received as a slightly yellow crystalline powder in a product yield of 78%.

Synthesis of H-D-Arg-D-Ala-*p*NA was performed by coupling of Boc-D-Arg-OH and H-D-Ala-*p*NA using benzotriazol-1-yl-oxytripyrrolidinophosphonium hexafluorophosphate (PyBOP) as coupling reagent (Figure S1, step C) (Frerot et al., 1991). For this purpose H-D-Ala-*p*NA was dissolved in dichloromethane (DCM) and the pH was adjusted to 8.0. The reaction mixture was cooled to -5°C and 1.2 eq. PyBOP, 1.05 eq. Boc-D-Arg-OH and 2.6 eq. *N*,*N*-diisopropylethylamine (DIPEA) were added. After stirring for 4 hours at -5°C the reaction was stopped by addition of water. Again the progress of the coupling reaction was followed by TLC. Extraction of the organic phase and removal of the Boc-protection group was performed as described above (Figure S1, step D). After vaporizing TFA, the remaining destillate was dissolved in ACN/water and purified via preparative HPLC. Product containing fractions were pooled and freeze dried. The product yield of this reaction step was 58%.

Finally, 2-aminobenzoic acid (Abz) was coupled to H-D-Arg-D-Ala-*p*NA by the addition of Boc-2-Abz-OH in the presence of PyBOP (Figure S1, step E). Conditions of the coupling reaction, equivalents of the reactants as well as the procedure for extraction and removal of the Boc-protection group correspond to those described above for the synthesis of the two building blocks. The crude reaction product Abz-D-Arg-D-Ala-*p*NA (Figure S1, step F) was finally dissolved in ACN/water and purified via preparative HPLC. Product containing fractions were again pooled and freeze dried. The final product was gained as a slightly yellow crystalline powder in ayield of 76% for this last reaction step. The overall product yield of the whole synthesis was calculated with 36%. Product identity and purity were proven via HPLC and LC-MS (Figure S2). The enatiomeric excess ee was found to be higher 99% as determined by enzymatic digestion of the compound with the L-Arg and L-Ala specific proteases trypsin and elastase, respectively.

2 Supplementary Figures

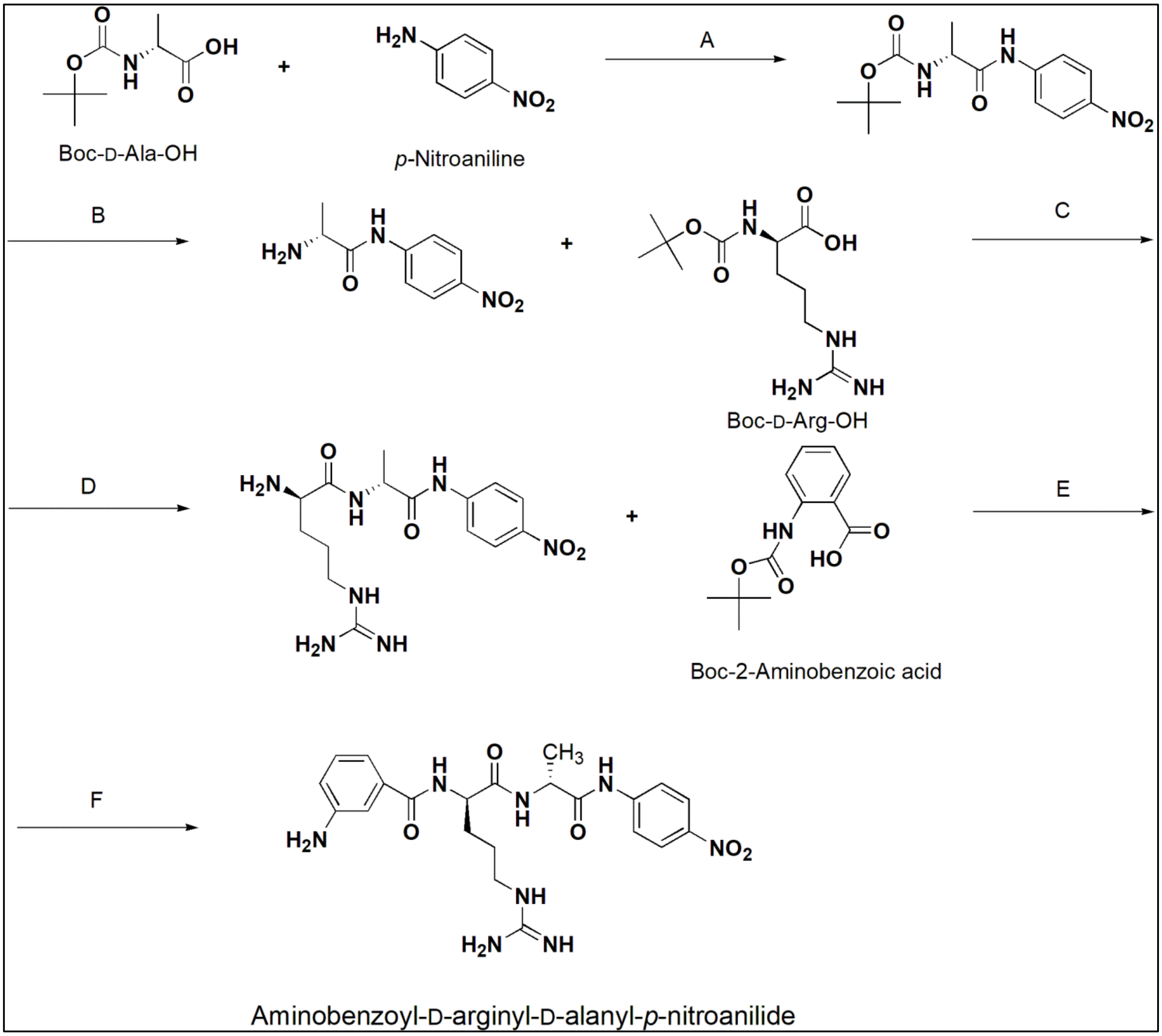

Supplementary Figure 1. Reaction scheme for the synthesis of Abz-D-Arg-D-Ala-*p*NA. A) Coupling of Boc-D-Ala-OH with 4-nitroaniline (*p*NA) via the method of mixed anhydrides, B) Removal of Boc-protecting goup, C) Synthesis of Boc-D-Arg-D-Ala-*p*NA by coupling of Boc-D-Arg-OH and H-D-Ala-*p*NA, D) Boc-deprotection, E) Synthesis of Boc-Abz-D-Arg-D-Ala-*p*NA and F) Boc-deprotection.

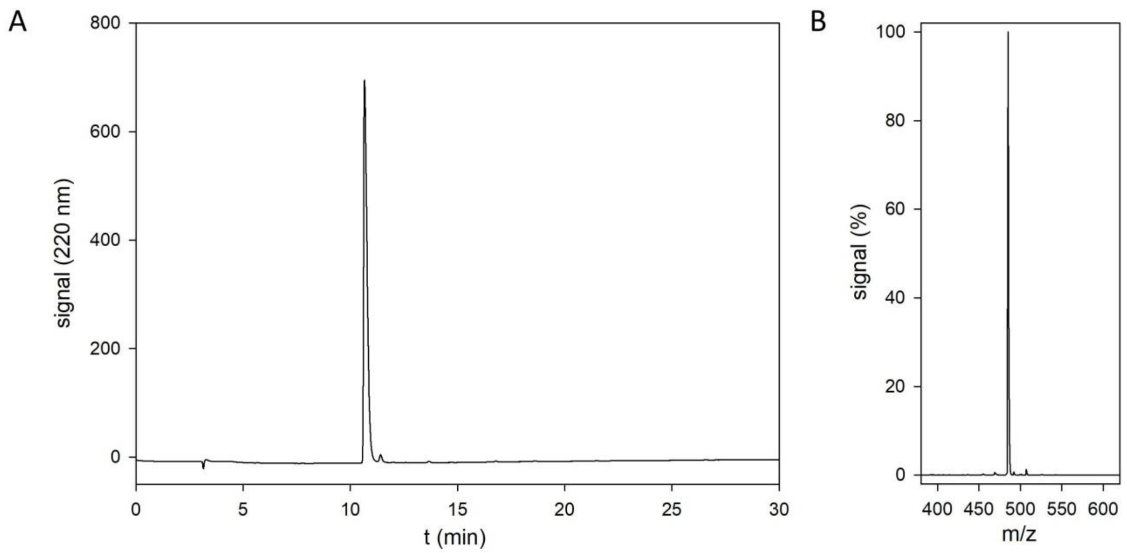

Supplementary Figure 2. HPLC- and MS-analysis of the synthesized substrate Abz-D-Arg-D-Ala-*p*NA. A) Analytical HPLC elution profile of the substrate via a continuous acetonitrile/water gradient from 0% to 100% acetonitrile in 30 min on a RP C18 column. Purity of the substrate is higher than 98%. B) ESI mass spectrum of the eluted peak: $m/z_{calc.}$=484; m/z_{found}=485 ($M+H^+$).

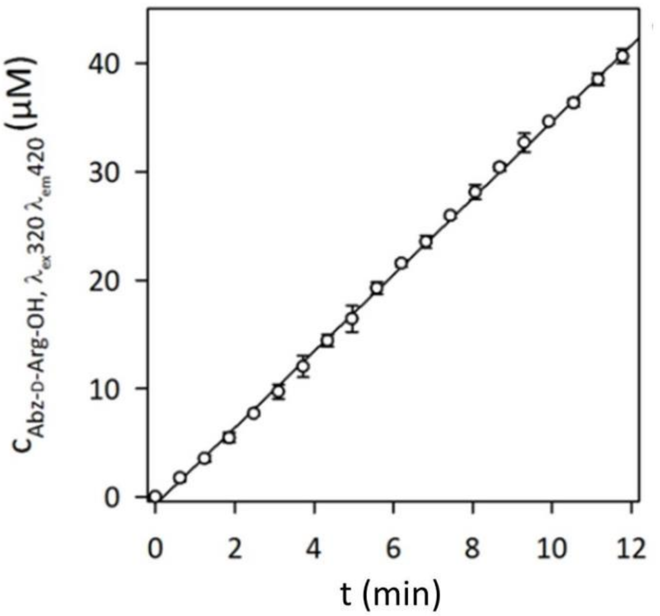

Supplementary Figure 3. Proteolytic activity of the purified D-Arg specific hydrolase towards the IQFS analysed via FRET-based fluorescence measurements. Conditions: 100 µM Abz-D-Arg-D-Ala-*p*NA, 50 nM enzyme, 100 mM phosphate buffer (pH 8.0), product formation was followed by fluorescence detection (λ_{em}=320 nm, λ_{ex}=420 nm).

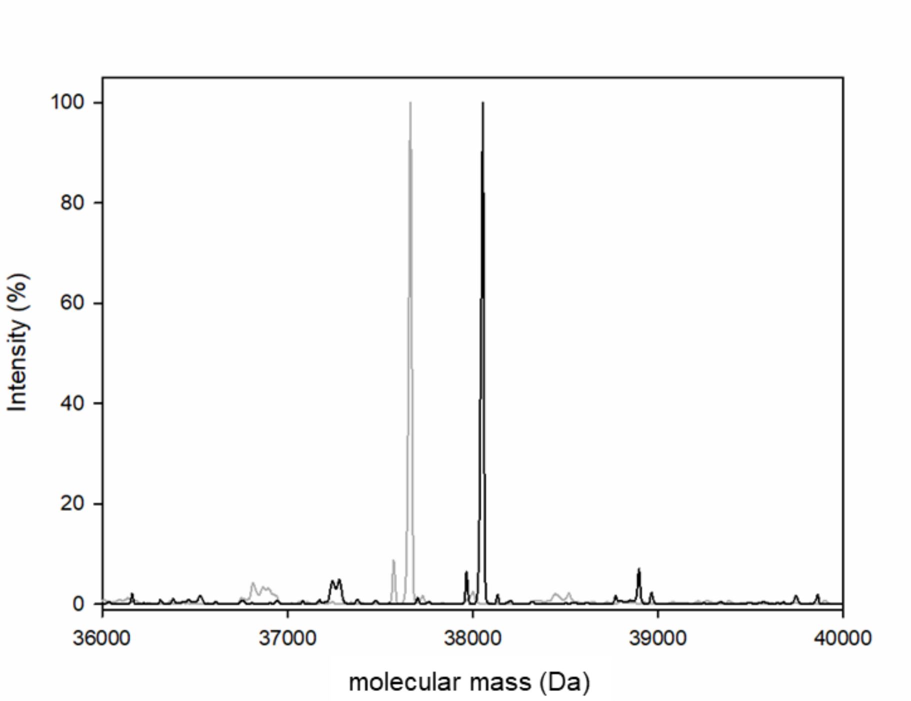

Supplementary Figure 4. ESI MS analysis of the D-Arg-specific DSP of *B. thuringiensis* in absence (grey) and presence (black) of ampicillin (amp). DSP (-amp): M_{found}=37,664 Da, DSP (+amp): M_{found}=38,014 Da. The difference between both molecular weights corresponds to the molecular weight of ampicillin (M=349 g/mol).

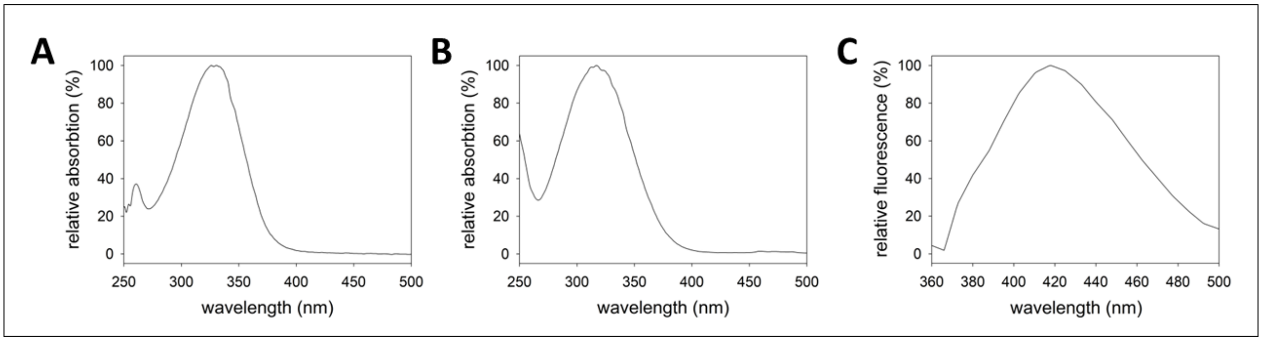

Supplementary Figure 5. UV/*vis*-spectra of the IQFS Abz-D-Arg-D-Ala-*p*NA prior (A) and after cleavage (B) by the D-Arg-specific DSP of *B. thuringiensis*. Both UV/*vis*-spectra have a maxium absorbtion intensity at a wavelength of 320 nm. Additionally the resulted fluorescence spectrum (C) of the cleavage product Abz-D-Arg-OH is shown with a maximum fluorescence intensity at 420 nm (λ_{ex}=320 nm). / Conditions: 100 µM Abz-D-Arg-D-Ala-*p*NA, 50 nM enzyme, 100 mM phosphate buffer (pH 8.0), reaction time 4 h. Analysis of educts and products was followed by UV/*vis*-analysis 250-500 nm and fluorescence detection (λ_{ex}=320 nm, λ_{em}360-420 nm).

Anderson, G.W., Zimmerman, J.E., and Callahan, F.M. (1967). A reinvestigation of the mixed carbonic anhydride method of peptide synthesis. *J. Am. Chem. Soc.* 89**,** 5012-5017. DOI: 10.1021/ja00995a032

Baumann, J. B. (1979). Solvent Selection for Recrystallization: Undergraduate Organic Experiment. *J. Chem. Educ.* 56(1), 64. DOI: 10.1021/ed056p64

Frerot, E., Coste, J., Pantaloni, A., Dufour, M., Jouin, P. (1991). Pybop and Pybrop: Two Reagents for the Difficult Coupling of the α, α-dialkyl amino-acid Aib. *Tetrahedron.* 47(2), 259-270. DOI: 10.1016/S0040-4020(01)80922-4

Lundt, B. F., Johansen, N. L., Volund, A., Markussen, J. (1978). Removal of t-butyl and t-butoxycarbonyl protecting groups with trifluoroacetic acid. Mechanisms, biproduct formation and evaluation of scavengers. *Int J Pept Protein Res*. 12(5), 258-268. DOI: 10.1111/j.1399-3011.1978.tb02896.x

König, W. and R. Geiger (1970). A new method for the synthesis of peptides: activation of the carboxy group with dicyclohexylcarbodiimide and 3-hydroxy-4-oxo-3,4-dihydro-1,2,3-benzotriazine. *Chem. Ber*. 103(7), 2034-2040. DOI: 10.1002/cber.19701030705
